# “Dark Corners”: Child Sex Murder, Forensic Expertise, and Protective Treatment in Socialist Czechoslovakia

**DOI:** 10.1093/jhmas/jraf011

**Published:** 2025-04-22

**Authors:** Andrea Bělehradová, Marie Láníková, Kateřina Lišková

**Affiliations:** University of Hradec Králové, Hradec Králové, Czech Republic; Masaryk University, Brno, Czech Republic; Czech Academy of Sciences, Prague, Czech Republic

**Keywords:** child sex offenders, Czechoslovakia, socialism, sexological treatment, sexual deviance, psychiatry, expertise

## Abstract

In 1966, the sexual murder of an eleven-year-old in Prague occupied the media and broader public, reinforcing expert debates about child sex offenders. In this article, we trace changes in medical experts’ understanding of child sex offenders in socialist Czechoslovakia between the late 1950s and mid-1970s. We show that psychiatrists and sexologists discussed forensic evaluation and the need for specialized treatment years before the 1966 case, but therapeutic practices lagged behind. As the networks of expertise shifted after this key crime, medical experts made up new kinds of people – deviant and non-deviant sex offenders – and gradually standardized treatment procedures. We argue that in addition to experts, the media and the lay public represented key agents who contributed to the establishment of a complex system of sexological protective treatment in Czechoslovakia. This system has remained almost unchanged to this day.

“Why did I have to get such a crazy sickness? If I had only known then where it might lead me, I would have insisted on some kind of operation in Bohnice. Why didn’t they do an operation? I keep searching in vain for an answer,” wrote 24-year-old Jaroslav Papež, accused of sexually abusing and murdering an eleven-year-old boy, from jail to his mother.^1^ In 1966, when Papež committed his crime, castration was newly legally available in Czechoslovakia. The law also allowed for protective treatment of sexual deviants. Despite Papež having committed similar offenses in the past, and despite being known to both law enforcement and psychiatrists, he was not dealt with as a dangerous sexual offender.

In this article, we explain why that was the case. We ask how did expertise on dangerous sex offenders against children emerge in state-socialist Czechoslovakia? How were the punishments and treatments of such offenders discussed? What were the roles of experts, lay audiences, and the state in that process? We show that a protracted expert debate about the nature of sexual deviance affected its legal framing and implementation. In the 1950s, psychiatrists and sexologists struggled over who had jurisdiction over sexual offenders, unable to agree on what terminology to use or who should forensically evaluate the people in question. Psychiatrists and sexologists in Czechoslovakia were medical doctors working clinically, with the main actors in these jurisdictional struggles also conducting research and publishing in specialized journals and university textbooks. We analyze how these two groups of experts gradually reached a consensus and how the law changed as a result. We highlight the ways in which everyday practice lagged behind this change, leading to the reality that people committing sexual offenses against children went unrecognized as dangerous, and were thus untreated. If they were sentenced, it was for misdemeanors such as disorderly conduct. A critical turning point was the case against Papež, which galvanized the public who were confronted for the first time with the reality of heinous crimes against children. The public and the media, in turn, demanded experts to account for their (in)activity. While this case did not launch forensic interest in sexual deviance, it propelled experts to formulate anew who was a child sex offender and what treatment was appropriate for him (because, in the expert view, it was always a “he”). From an inconspicuous man groping children who, if interpellated by law enforcement at all, would be charged with a misdemeanor and given a suspended sentence, the experts made up a dangerous child sex offender who should receive the death penalty (or at least a maximum prison sentence with a subsequent sexual protective treatment). We argue that the processes we analyse have a much stronger impact than mere redefinition. Indeed, we show how a new category of person, a child sex offender, was made up by experts where no such category had existed before. These offenders were exclusively men, the victim in this pivotal case was a boy, and the majority of experts involved were also male; we ask what role gender played in the process of identifying and treating sex offenders.

We focus on medical expertise, specifically in the fields of forensic psychiatry and sexology, and the changes it underwent in understanding sex offenders against children between the late 1950s and mid-1970s. We use a wide range of contemporary medical and criminological journals, scholarly monographs on forensic psychiatry, government reports on psychiatric care, and expert seminars on sexual deviance unearthed in the National Archives of the Czech Republic. To capture the reactions of the general public to the sexual murder of a boy in 1966, we examine letters sent to the most widely read magazine, *Vlasta*. Some letters were published in the magazine, and we found others in the archives of the Czechoslovak Women’s Union, *Vlasta*’s publisher.

To make sense of the shifts in expert understanding and treatment of sex offenders against children, we draw on Gil Eyal’s theoretical framing of the sociology of expertise. We understand expertise as “a network linking together agents, devices, concepts, and institutional and spatial arrangements.”^2^ Using this approach, we reveal “a new institutional matrix within which a new set of actors [in this article psychiatrists, sexologists, media, and the general public] were able to forge an alternative network of expertise.”^3^ The sociology of expertise helps us analyze the jurisdictional struggles between different groups of experts (mainly psychiatrists and sexologists) over forensic assessment and the treatment of sex offenders, and the ways their expertise intersected and resulted in new knowledge. For our analysis, networks of expertise are more important than individual experts.

Since at least the beginning of the nineteenth century, different kinds of experts were preoccupied with questions of guilt and responsibility for a crime: who is and who is not responsible for a criminal deed and how each category of perpetrators should be treated. Experts – criminologists, penal reformers, forensic evaluators and others – debated the issues of free will, social and biological conditioning, and protection of society.^4^ Importantly for our argument here, expert knowledge was produced and changed over time, and with it the ideas about offenders and their most appropriate treatment continued to evolve.

Following the work of Ian Hacking, we show how new kinds of people – sex offenders against children – come into being through the realignment of systems of knowledge. In Hacking’s words, “a kind of person came into being at the same time as the kind itself was being invented.”^5^ As experts refined the understanding of sex offenders, they simultaneously made up new kinds of people. New kinds of treatments then emerged to manage the expert understanding of the new categories of people.

When experts make up a new kind of person, the new category needs to be named, and the people within it labelled accordingly. Czechoslovak experts over the course of the 1960s and the 1970s gradually acknowledged that even though someone committed a sex offence – and could be labelled as a sexual delinquent – it did not mean he would also be recognized as a sexual deviant and vice versa. However, the two groups – sexual delinquents and sexual deviants – often overlapped and mingled in forensic and treatment praxis, especially because sexual deviants were usually referred to experts only after they had committed a sexual delict. The experts from the sexological ward of the psychiatric hospital in Horní Beřkovice, which was founded in 1974 as the first in the country to provide sexological protective treatment, wrote shortly after its launch:

[Sexological] patients only rarely tend to be true sexual deviants. They are usually sexual delinquents in the broadest sense. Thus, psychopaths, alcoholics, organics (demented), and schizophrenics – along with otherwise “normal” sexual deviants – meet here side by side. Dividing these individuals into groups and channeling their interaction is a problem in itself. The issue of a narrower division – according to types of deviant behavior – seemed extremely superstructural to us in the given situation.^6^

The line dividing sexual deviants from sexual delinquents was tenuous, even for sexologists. In this article, we use the labels “sexual deviant” and “sexual delinquent” as the Czechoslovak experts themselves recognized and used them.

## Eastern European Expertise on Sexual Deviants and Delinquents

Social science research on expertise and its role in shaping the understanding of sex crimes and treatment of deviants in socialist Czechoslovakia has flourished in recent years. Examining sexological research on homosexuality in Prague during the 1950s, Kate Davison has shown the crucial involvement of Czechoslovak experts in decriminalizing homosexuality.^7^ Although supported by the socialist government, medical scientists were inspired by more than the state’s approved Pavlovian teaching and incorporated psychoanalytic views. In fact, as Sarah Marks has shown, psychiatry in socialist Czechoslovakia “was afforded more autonomy than many other fields in intellectual endeavour” in the country.^8^ There was, in other words, a “remarkable level of plurality” in Czechoslovak expert thinking in the post-Stalinist years, starting from 1957.^9^ As Davison has argued, based on psychoanalytic concepts, the sexologists did not consider sexual orientation or the psychosexual structure of personality to be rigid. Influenced by Pavlovian teaching, they treated sexual deviation through a method of conditioned reflexes.^10^ The sexologists concluded that the method could affect a person’s sexual behavior; for example, a homosexual man could get used to heterosexual intercourse and get married. However, they acknowledged that the treatment might not be the solution that homosexuals needed, as the problem lay in a lack of social acceptance of homosexual behavior.^11^ As a result, sexologists called for the decriminalization of homosexuality, which was legally codified by the state in 1961.^12^

Although Czechoslovak experts were studying homosexuality in the 1950s, other kinds of sexual deviations and delinquency remained off their radar. Kateřina Lišková showed that in the 1950s when the focus on work was paramount, sexual deviants remained invisible to experts unless their sexual behavior interfered with their work status and work ethic or harmed the state’s economy.^13^ After the failed attempts at political reforms known as the Prague Spring, “[t]he mode of subjection shifted from work to family.”^14^ Anyone who did not fit into the traditional family model and failed to establish and/or maintain a heterosexual relationship could be deemed suspicious during the period known as “Normalization” in Czechoslovakia (1969–1989). As a consequence, the number of men labelled as sexual deviants rose, and experts called for specialized inpatient sexological care. As sexologists helped the state in promoting traditional gender and family policies, their cultural authority in turn was strengthened, leading to sexology’s institutionalization in 1974.^15^

There was, in other words, a close alliance between Czechoslovak sexologists and the socialist state regarding care for sex offenders, as Kateřina Lišková and Andrea Bělehradová have shown.^16^ Therapeutic castration, for example, became an almost indispensable part of the state treatment for “dangerous” sex offenders. In turn, sexologists gained authority in treating sex offenders during socialism. Their forensic and clinical expertise in treating cases of sexual delinquents has largely remained undisputed to this day. Although therapeutic castration was significantly reduced after repeated criticism by the Council of Europe in the 2010s, the treatment of sexual deviance has remained almost unchanged in the Czech Republic (formerly Czechoslovakia) since its establishment in the 1970s.

Sexological classification and the treatment of sexual deviants are not the only things that underwent fundamental changes during socialism. As Kateřina Lišková and Lucia Moravanská showed, the criminological understanding of spousal murder also transformed rapidly in accordance with shifts in state politics.^17^ In the 1950s, experts explained these crimes in terms of the lingering ills of the preceding political system and the structural conditions of society – class origin discrepancies between spouses or a bourgeois way of life of the perpetrator. During the period of Normalization, criminologists favored an individualized explanation, attributing the causes of such crimes to the psychopathological personality of the killers. With the increasing emphasis on the private sphere after 1968, the regime and its experts disregarded the possible causes of spousal murders resulting from broader social structures. Lišková and Moravanská have termed this “the psychiatrization of criminological expertise,” which supported individualized explanations of spousal murders by searching the murderer’s background for a dysfunctional family environment and psychopathological features.^18^ Czechoslovak experts depicted spousal murders as emotional crimes; the killers were described as femininized and therefore seen as presenting “a lesser social danger” than other types of deliberate murderers during Normalization.^19^

By contrast, sexually motivated child murders caused a great uproar among experts and the general public in the late 1960s. Jakub Střelec explained that through the new laws codified in the early 1960s, the state sought to eradicate “antisocial parasitic elements” from the socialist society.^20^ To meet this goal, the state gradually relied on the work of its experts – mainly criminologists and psychiatrists. Sex offenders attracted prominent expert attention because psychiatrists, psychologists, sexologists, and courts disagreed on the assessment and punishment of these offenders. Střelec identified the case of Jaroslav Papež as the turning point that “marked the beginning of a long discussion among experts and state agencies about the most effective way of treating and handling sexual offenders.”^21^ We agree with Střelec that the crime provoked a tremendous public response. In this article, we detail the extensive media coverage and point out a pivotal role played by one journalist devoted to women’s and children’s issues. However, we also challenge Střelec’s argument by showing that the expert debate about forensic assessment and specialized treatment of sex offenders had already started years before Papež’s crime. We suggest that Papež’s offense and the subsequent public outrage amplified existing expert efforts to develop more systematic measures for assessing and treating sex offenders, which resulted in opening the first psychiatric ward specialized in sexological protective treatment in the mid-1970s.

Compared to socialist Czechoslovakia, there has been little research about expertise centered on sex offenders in other socialist East-Central European countries. Greg Eghigian has shown that in the 1950s the East German state deemed sexual perpetrators to be weak-willed, immature, and unstable psychopaths, unable to control their impulses and desires.^22^ The psychopathy diagnosis included the assumption that the offenders were incorrigible. The idea of corrigibility appeared in the 1960s, when criminology, forensic psychology, and the criminal justice system in East Germany started finding sex offenders to be “men requiring civilization in the form of modern, scientifically based treatment and therapy.”^23^ They saw sex crimes as relics of the past, as failed processes of socialization that could be solved – not by harsh punishment, but by efforts to bring the perpetrator back to the right path. The question of soundness of judgment became crucial in cases of sex offenders and with it the role played by psychiatrists. Psychiatrists assessed the possible corrigibility of sex offenders’ personality and offered a social prognosis that helped to decide between prison and a psychiatric hospital.

Examining sex crimes in socialist Poland, Agnieszka Kościańska has shown that various experts (sexologists, educators, lawyers, and journalists) started to discuss the crime of rape in the 1970s.^24^ When a woman was raped, sexologists and legal scholars elaborated on her responsibility for the crime and assessed her moral behavior (recklessness and provocation) and family background during the trials. These practices were in accordance with Polish ideas about traditional gender roles, which scientific experts stressed as vital for sexual satisfaction and harmonious families in the 1970s. Polish experts perceived women’s sexuality as driven by feelings, and male sexual desire was deemed to be unrestrained because of their different hormonal system. Therefore, experts held women responsible for directing male sexuality and co-responsible for sex crimes as a consequence.

In Czechoslovakia, experts discussed the issues of guilt, self-control, and treatment in relation to adult sexual offenders targeting children. In the following sections, we first interrogate forensic psychiatrists’ deliberations on guilt vis-à-vis a person’s diminished sanity (*zmenšená příčetnost*), and detail sexologists’ struggles with psychiatrists over who had the jurisdiction to offer qualified forensic expertise in cases of sex offenders. We show that these debates took place at a time of impoverished psychiatric inpatient treatment. Obsolete spatial arrangements in psychiatric hospitals presented another obstacle to conceiving of efficient care for patients with sexual deviations. We identify when these two groups of experts found a common language, paving the way to the experts’ ability to forge a new network of expertise. After describing the murder committed by Papež, we consider the public outrage which commenced in the pages of a high-circulation magazine. We focus on the role of the editor who curated letters from readers and elicited expert responses. In the final section, we present how psychiatrists and sexologists smoothed the remnants of disagreement in their respective expertise and presented their classifications of sex offenders – a type diagnosed with a sexual deviation versus a type characterized as non-deviant – to criminologists, and how the state finally reacted by issuing a series of guidelines and regulations that launched the protective treatment of sex offenders that exists in the Czech Republic to this day.

## Jurisdictional Struggles between Sexologists and Psychiatrists

In the 1950s, Czechoslovak doctors paid little attention to sexual deviants. If forensically assessed, it tended to be psychiatrists rather than sexologists who were invited as forensic experts and who had jurisdiction over the issue of men committing sex acts against children. For example, psychiatrist Ivan Horvai was a frequently invited forensic expert who later also provided testimony in the 1966 sexual murder case of Jaroslav Papež. Two features were characteristic of the forensic expertise provided by Czechoslovak psychiatrists in the 1950s. First, they rarely mentioned the sexual aspects of psychiatric diagnoses. Second, when evaluating people who committed a crime, psychiatrists struggled with assigning diminished sanity to perpetrators, which had clear consequences for sentencing.^25^

First, psychiatrists paid rather little attention to the sexual proclivities of people with diagnosable psychiatric conditions. In the 1957 book *Forensic Psychiatry for Lawyers and Medical Doctors*, psychiatrists Ferdinand Knobloch and Jiřina Knoblochová discussed sexuality in terms of sexual needs, which they argued had inherited, congenital, and social roots. According to the authors, human need might be disturbed and there was always a continuum from normal to perverted needs (*úchylné potřeby*). While normal people might also feel perverted needs, they did not act on them. Certain pathological states were accompanied by either increased or decreased sexual needs. The authors mentioned dementia as a state in which “inhibitions fall away, and here the sexual urge may manifest itself as morbidly heightened. The vague desire is not directed toward a definite goal, but manifests itself, for example, in exhibitionism, stimulating the genitals of small children, etc.”^26^ The authors also focused on oligophrenia (feeble-mindedness), and especially its moderate forms in which people “easily commit sexual offenses (exhibitionism, fornication by force).”^27^ Thus, both dementia and oligophrenia involved diminished intellectual capacities that could prevent a person from controlling his perverted needs. Or a man might be a psychopath and a perverted sexual need could be a major trait in his psychopathic personality, such as in cases of exhibitionism (specifically, grown men flashing their genitals in front of little girls) or homosexuality. The authors admitted that little was known about where these proclivities came from or how to cure them.^28^ Other than a few specific examples, Knobloch and Knoblochová did not elaborate on sexuality in forensic evaluation.^29^

Czechoslovak psychiatrists in the late 1950s understood sexual offenses as typically committed by psychopaths. When psychiatrist Ivan Horvai discussed psychopathy in 1956, he mentioned “rare, but nonetheless existing sexual perversions” – but, confusingly, identified these perversions *in* children, not as committed *against* them, and suggested their treatment in “strong healthy children’s collectives.”^30^ “Virtually the same applies to adult psychopaths,” was all that Horvai had to say about grown-ups, yet his statement related more to general treatment than to the sexual components of their conditions.^31^ In an article published one year later, Horvai wrote about 54-year-old G. who was accused of sexual abuse and indecent acts.^32^ G. was diagnosed with polymorphic psychopathy and cerebral arteriosclerosis – and as such his evaluation combined both psychopathy and dementia, two distinct diagnoses that could lead to committing a sexual crime.

Indeed – and that goes to our second point – the case of G. represented just one example of a problem psychiatrists often encountered in their forensic practice: diminished sanity and insanity. Horvai noted that G. was evaluated as having diminished sanity (*zmenšená příčetnost*), “but to a lesser extent than intended by §21” of the Criminal Code. Overall, psychiatrists were persuaded that “it would be wrong to punish the mentally ill, but we need to treat them.”^33^ But were those who committed a sexual misdeed mentally ill? Knobloch and Knoblochová tended to respond in the negative. The sexually deviant (*pohlavně úchylní*), typically men, might suffer from a mental disorder but if so, “it is not usually of such a degree as to cause a state of insanity or near insanity.”^34^ To Czechoslovak experts in this period, the label of psychosis was the only clear indication to forensically evaluate and rule the deed as committed in a state of insanity or at least diminished sanity. When defining “[m]ental disorders most frequently leading to insanity,” psychiatrists mentioned first and foremost “mental illness” (*duševní choroby*), defined as “mental disorders of longer duration, characterized by a more or less typical beginning, course, and eventual end: progressive paralysis, senile dementia, schizophrenia, manic-depressive psychosis, and others.”^35^ Such conditions were seen by experts to trigger psychotic states during which a person had no control over their actions. With proper psychiatric care, psychosis was seen as treatable. Psychopathy, on the other hand, was viewed as neither treatable nor grounds to exonerate a perpetrator on the basis of insanity.

Pondering the etiology of psychopathy in 1957, Knobloch and Knoblochová identified the social regime as the culprit:

The widespread problem of psychopathy is one of the by-products of the capitalist order. Only in a socialist society does the problem of psychopaths cease to be a widespread social problem. This does not mean, however, that one should wait and not try to remove the vestiges of the past through re-education and also through treatment. For the time being, psychiatrists, because of the lack of facilities, have little opportunity to help solve the problem of psychopaths from the therapeutic side.^36^

The psychiatrists pointed to larger forces beyond medical help, called for medical treatment, and indicated the institutional impossibility of such treatment all in one short paragraph. The conundrum became even more pronounced when Knobloch and Knoblochová added punishment to the equation: “It is clear that for some [adult offenders] the threat of punishment does not provide sufficient motive for re-education and they commit new crimes over and over again. This is particularly striking with the sexually deviant, homosexual, and exhibitionist offenders.”^37^ They concluded, “current advances in psychiatry justify the hope that in the future psychiatry will contribute its modest share to strengthening the educational effect of punishment and will also make it possible to replace punishment for some crimes (for example, some sexual deviations) with treatment.”^38^ The ambivalent situation in which psychiatrists viewed psychopathy as untreatable, yet in need of treatment that remained unavailable, was further exacerbated by disagreements in diagnosis and forensic evaluation among psychiatrists and between psychiatrists and other experts.

Psychiatrists disagreed among themselves because at the time attributing a psychiatric diagnosis was itself a less than straightforward matter. Evaluating a person’s state of mind in light of such a diagnosis was even more difficult. As Horvai described the state of things in 1957,

different experts have assessed the perpetrator’s liability in different ways – seemingly contradictory, according to the judicial authorities. However, when their opinions were compared, it turned out that they had almost the same opinion on the mental state of the perpetrator – but one of them considered that this state could not yet be classified as insanity and was rather a state of diminished sanity, while another thought that it was perhaps a state that satisfied the requirements of §21 of the Criminal Code, and yet another considered it to be a state of quasi-normality, etc.^39^

A possible solution to this disunity might be introducing “degrees of diminished sanity” to the law, as Horvai had noted in case of G. whose diminished sanity was further qualified as “to a lesser extent than intended by §21.”^40^ Forensically evaluating sanity in percentages, instead of a binary of sane or insane, would thus gloss over disagreements within the discipline of psychiatry.

However, the psychiatrists perceived insurmountable problems in forensic evaluation when intruders from other, non-psychiatric disciplines presented their expert opinions. According to §124 of the Criminal Procedure Code, “If it is necessary to examine the mental state of the accused, two experts, preferably psychiatrists, shall always be brought in.”^41^ The formulation “preferably” opened the door to invite other experts, not exclusively psychiatrists – which was a thorn in the side of psychiatrists. “Assessments by non-experts who do not know psychiatry as a whole and do not work in psychiatry can often lead to incorrect or distorted conclusions,” insisted Horvai.^42^

Sexologists were chief among the intruding experts. However, sexologists also paid little attention to sexual deviance in the 1950s. In the otherwise vast clinical and research practices of Czechoslovak sexologists in that decade, sexual deviance was hardly present; the low numbers of deviants were reflected in the low number of scholarly articles about deviance and/or delinquency, as compared to male and female infertility.^43^ At the end of the 1950s, two articles were published by sexologists devoted to “Some findings of the assessment of sexual delinquents from the sexological point of view,”^44^ and to “The question of sanity and corrective measures for sexual delinquents.”^45^ In these articles, sexologists lamented that judges were not asking their professional opinions, and instead preferred assessments from psychiatrists, whose work the sexologists criticized for ill-informed conclusions. As Karel Nedoma and Kurt Freund argued in 1959,

Cases of sexual delinquency and expert investigations and assessments of offenders constitute a border area of psychiatric forensic work. The usual experiences of the experts from the psychiatric practice are in many cases not sufficient, especially when deeper knowledge from the field of sexology is also desirable. The cooperation of security authorities [i.e., police] and courts with medical experts in the assessment of sexual delinquency is unsatisfying. The cooperation is completely inconsistent, haphazard, and without the necessary scientifically grounded criteria.^46^

In an effort to demonstrate the current state of the art in the forensic processes in Czechoslovakia, Nedoma and Freund presented a set of information about all the sexual delinquents they had evaluated in the previous year (in 1958). Their data probably represent the only publicly available medical statistics about sexual delinquency in Czechoslovakia in the 1950s. Out of – according to the authors – “only” 24 men, pedophilic activities were the most frequent, with sixteen perpetrators (ten of them heterosexual and six with a homosexual object); other forms of misconduct were represented only sporadically.^47^ As the authors explained:

It is very reasonable from a legal point of view that the most common occurrence in our forensic assessments were pedophilic activities, whose severe social danger is evident and reflected also in the length of penalty. However, our experience has shown that the way the courts have cooperated with experts has so far been largely haphazard. From an expert point of view, it is not possible to anticipate that sexual abuse or other sexual delicts could be committed by an individual who is fully healthy mentally and adequately developed sexually. Nevertheless, the cases in which some psychogenic binding reactions were randomly manifested, including rarely more serious mental disorders, were mostly submitted to us for assessment.^48^

Looking for an underlying pathological cause to such sexual conduct, the sexologists examined the mental state of the offenders. They recorded mental deterioration on the basis of arteriosclerosis in five cases, oligophrenia four times, psychopathy and other anomalies eight times, and neurosis in one case. The sexologists did not find any pathological deviation that could be identified as the singular source of the delinquent behavior. Nonetheless, they ruled out the possibility that sexual delicts “could be committed by an individual who is mentally quite healthy,” and as such opened the door for lenient sentencing on account of mental illness and diminished penal responsibility as its result.^49^

With problems in understanding the etiology of sexual deviance, diagnosing, and providing (forensic) evaluation, it comes as little surprise that options for treatment were also deemed insufficient. As psychiatrists Knobloch and Knoblochová noted, “so far the psychiatric hospitals are not equipped for treating cases sent by the courts.”^50^ But when state controllers visited every psychiatric hospital in Czechoslovakia at the end of the 1950s, the bleak conditions of psychiatric care became apparent. Psychiatric facilities suffered from material deficiencies and a lack of personnel. In century-old buildings, dozens of beds were crammed into the rooms where patients spent most of their time as day-rooms and dining halls were missing. Patients ate in corridors, using tin bowls and spoons, often while sitting on the floor. Basic hygienic necessities, such as soap or toothbrushes, were not provided, and up to eight patients shared a sheet in lieu of a towel to dry themselves after a rare shower.^51^ Pharmacotherapy was also inefficient and dispensed medicine was not logged correctly.^52^ Qualified personnel were lacking: there were not enough doctors or nurses, and even custodial staff were in short supply.^53^ Patients were often ordered to do the work needed on the premises, which amounted to “abusing the ill.”^54^ This was compounded by the absence of patients’ rights, including of those who were sent by the courts.^55^ The controllers also lamented the underdeveloped psychiatric knowledge, as compared with the Soviet Union or capitalist countries. “Psychiatry as a science is still in a preliminary orientation phase,” stated the fifth report. Diagnostics were deemed as “imperfect,” “disintegrated,” and “relying on symptoms without understanding underlying causes.”^56^ The reports called for scientific cooperation across national borders and increased collaboration among various ministries with the aim of dramatically improving psychiatric care and issuing binding guidelines for court-ordered detention.

## Finding a Shared Language between Psychiatrists and Sexologists

In 1959, the sexologist Nedoma and the psychiatrist Freund criticized the absence of scholarly cooperation and called for specialized treatment of sex offenders.^57^ In May 1960, the first seminar took place that focused solely on the forensic aspects of sexual deviations. The psychiatrists and sexologists who gathered in Hradec Králové noted that, “In cases of clearly socially dangerous sexual activities, current penal measures do not deal rationally with either the issues of correction or the protection of society. The usual measures in the majority of cases do not accomplish more than a temporary isolation of offenders whose dangerousness persists without substantive changes even after their release.”^58^ Participants suggested differentiated treatment of sex offenders and – in cases of “gravely asocial sexual deviations where a person himself requests it” – the experts proposed castration.^59^ Invoking favorable results reported from abroad, this method was to be legalized in Czechoslovakia and prescribed “in individual cases by a commission comprised of a lawyer, a psychiatrist, a sexologist, and an endocrinologist.”^60^ In other cases where treatment outcomes remained uncertain, experts suggested “special measures for protecting society that would harm the perverted individual as little as possible,” namely special institutions for long-term or even life-long detention. The final communiqué of the seminar called for establishing such institutions and inviting a psychiatrist as a forensic expert in all court cases dealing with sexual deviations.^61^

While protective treatment in some form existed, its practice was flawed. As a government investigation found in December 1960, many psychiatric facilities did not report on the course of protective treatment to the courts that had sent the individuals in the first place.^62^ These procedural deficiencies and a newly established expert consensus informed the new Criminal Code of 1961. Its §72 stipulated that protective treatment (*ochranné léčení*) could be ordered by the court for those dangerous perpetrators whose freedom of movement was dangerous to society, yet whose insanity contradicted imprisonment. Protective treatment was to take place in a healthcare institution, lasting “as long as is required,” and only the court could decide about the termination of treatment.^63^ Moreover, §25 allowed for “refraining from imprisonment if the perpetrator committed the crime in a state of diminished sanity.”^64^ As forensic psychiatrists acknowledged, the new law “extended protective treatment to the diminished sane, decoupling diminished sanity from mitigating circumstances.”^65^ Indeed, diminished sanity was no longer used to excuse the perpetrator and his deed, but rather to send him in a protective facility. In instances of sexual deviance, by law a perpetrator had to be evaluated as having committed the deed in a state of diminished sanity and to have his future danger evaluated as likely – only then could he be sent into protective treatment.^66^ Yet as these psychiatrists noted in 1965, facilities specializing in such treatment did not exist.

With inpatient protective treatment not yet available, two other treatment options –adaptation therapy and surgical castration – were implemented for convicted sexual deviants and/or delinquents in Czechoslovakia in the mid-1960s. Nedoma introduced the first change in treating sexual deviants in 1964 when he designed “adaptation therapy.” This treatment consisted of two phases involving outpatient psychotherapy accompanied by a hormonal adjustment. The first “attenuation phase” lasted for several months and involved a “rapid and profound reduction of androgen activity” through the administration of hormones to ensure a decrease in the deviant sexual desire and thus prevent a relapse of his sexual criminality.^67^ During this phase, a sexologist sought to establish a trusting therapeutic relationship with the patient. In the second “maintenance phase,” the dosage of hormones was reduced, and the patient attended psychotherapy sessions (at least three times per week) during which the sexologist guided him to “adapt to normal forms of sexual expression.”^68^ As Nedoma stressed, a “sufficiently deep and fixed patient motivation for sexual deviation treatment” was crucial for the therapy to be effective.^69^ However, motivation and effort to deal with deviant sexual desires were lacking in many sexual delinquents, which complicated the treatment and diminished its results (measured in sexual recidivism statistics).^70^ Perhaps it was the delinquents’ lack of effort to cope with and avoid acting on deviant sexual desires that led the authorities to introduce the second change by legalizing surgical castration just two years later, on 30 March 1966.^71^ At the time, Czechoslovak experts still considered the procedure to be a last resort, available only exceptionally to the most dangerous sex offenders.^72^

## The Breakthrough Case of Jaroslav Papež

On 5 June 1966, eleven-year-old Josef Neumann stood in the passage of the Metro Cinema in Prague’s city center.^73^ He was eyeing the poster for a detective movie that was age restricted. It was not difficult to accept an offer from a man who said he was a projectionist at the Metro to watch the film from the projection booth. Papež led Josef inside the booth and ordered him to get undressed. Then he started groping him and cutting his hair. With the haircut, Papež realized it would be impossible to explain what had happened and he started stabbing the boy with scissors, and eventually strangled him to death.

Papež was not new to the sexual abuse of children. Since he was 20 years old, he had amassed a lengthy criminal record. He was first investigated in 1962 for groping a ten-year-old boy, then again in 1963 for undressing and groping a twelve-year-old boy whom he also stabbed with a needle. A mere ten days after his second investigation he undressed another ten-year-old boy and tried to strangle him. He was sentenced in 1964 to one year in prison with a three-year suspended sentence and ordered to undergo treatment for alcoholism. This institutional treatment was soon changed to outpatient treatment. Within a month of his release, he had flashed his erect penis in front of pre-pubescent girls and undressed and groped another ten-year-old boy. He was investigated and placed for examination in a psychiatric hospital in Bohnice. In April 1966, he was sentenced for disorderly conduct (*výtržnictví*) to eight months in prison. He was supposed to report to prison in the week of June 6. He committed his final crime one day before he was to report to prison.

After Josef’s dead body was found four days later, decomposing in the cinema’s air shaft, the police knew whom to look for. They arrested Papež, who immediately confessed his crime. Two forensic experts were invited to evaluate the perpetrator. Psychiatrist Ivan Horvai claimed that Papež was sane. Sexologist Kurt Freund held that Papež was insane and thus was not responsible for his actions. And then the public and the women’s magazine *Vlasta* weighed in.^74^

## Public Outcry Over Child Sex Murders and Abuse

The public’s reaction to Papež’s crime was clear – he must be executed. The court received a petition signed by 860 citizens demanding Papež’s death. Finally, Papež was hanged on 8 June 1967 – a year after his crime.^75^

In October 1966, the women’s magazine *Vlasta* published the first article in a three-part series titled “Dark Corners” devoted to the sexual abuse and murder of children. The series opened with a text by the editor Věra Hájková, who stressed the ineffectiveness of existing punishments and the absence of detention centers for perpetrators.^76^ Hájková emphasized that the fear of a severe sentence did not discourage offenders from repeating their crimes.^77^ In addition to Papež, she recounted other instances of sexually motivated brutal acts against children, including the murder of a four-year-old girl.

The public outcry against the sexual murders was enormous. The editorial office received many letters from readers. The letters were sent by parents – mothers as well as fathers – but also by people whose parental status was not identified.^78^ Most of the letters could not fit in the pages of the magazine but were kept in the archives of the Czechoslovak Women’s Union, *Vlasta*’s publisher. In the letters, readers pondered the options for protecting children, asked authorities to intervene, and offered to help in establishing detention centers. Even though censorship was not officially abolished until June 1968, political relaxation and a softening of the regime began to take place as early as 1963.^79^ In the context of this liberalization process, *Vlasta*’s editor Hájková used the opportunity to raise a topic that could point to the shortcomings of the socialist establishment. At the same time, citizens were not afraid to send letters demanding an immediate remedy, including tougher penalties for sexual abuse. Hájková thus brought the demand for more control and inpatient treatment of sexual offenders into the public arena.

*Vlasta* published a few of the letters, and Hájková strategically selected those that supported her argument for publication. As a skilled editor, she opened with the personal experiences of affected parents. She continued with the example showing that warning small children was never enough. Hájková then devoted the most space to those letters demanding “long-term or permanent isolation of sexually deviant people” and castration which, as she pointed out, was called for by the majority of letter writers.^80^ The narrative that Hájková presented and illustrated with examples was clear: the cases of sexual abuse and murder were extremely disturbing. It is not possible to effectively warn children, and the only reliable protection of children means isolating the sexually perverted offenders (*úchylný*).

According to Hájková, the most horrible letter came from a mother whose son had been a victim of a perverted man:

No one will return my boy to me, which makes my pain and indignation all the greater. These disturbed people are not to be amnestied in any way – this happened in our case; if he had not been amnestied, our boy would have lived! [...] There is nothing I can do; my life has been marked. Even though I have one more child, I can no longer be satisfied. And that is why I would like everything to be done so that no mother will have to suffer like this again.^81^

According to this upset mother of a murdered nine-year-old boy (murdered in 1963 in Pardubice), the death penalty for any murderer was too late as the child’s life could not be saved.

A mother from the town of Mýto pointed out that the youngest children were most in danger, as they could not understand the risks: “I have a 3-year-old daughter [...]. She is not afraid of strangers, whoever speaks to her and holds out their hand, she goes with them. I don’t leave her alone outside, but I’m afraid of what could happen later.”^82^ The readers – usually parents of small children – were outraged by light sentences for sexual assaults against children. Among the letter writers was a father of four children from Písek who wrote, “Therefore, I approve of the requests of other writers that a short process be made with such perverts: one tort, a heavy and long sentence, the second one, the rope without mercy. No waiting until such a coward destroys life and even deprives a child of life. A child who has not yet had and cannot have any idea of such things at their age.”^83^ According to the editor, it was mainly because of the detailed information about the murder in the Metro Cinema committed by Papež that was written about in the popular press that some readers could react, and they did so expressively. For example, a writer from Prague proclaimed, “I would not kill a chicken myself, but I could kill a man who would do something like that to my grandchild. Just recently, when the little girl’s tragedy happened, and people from the whole village went looking for him with pitchforks and rakes, the police prevented it. Why didn’t they let them avenge the little girl?”^84^ Similarly to the other writers, this author was shocked by the light sentences, the lengthy legal proceedings, and the lack of prevention. Likewise, one woman from the village of Stonařov noted in 1966,

It is monitored whether a biker is wearing glasses, but what such a creepy guy does in his free time, a guy who lives next to the police, does not interest the comrades anymore. We are a cool people, like the English, I guess. But when something happens, there are a few dead bodies and people get involved, then something is done. Better late than never. And then it is quiet again. Amnesty comes, and the guy is out. Nobody notices him again until he does something.^85^

As Hájková pointed out, the punishments were light in contrast to penalties for thefts of public property. But according to psychiatrists and sexologists, the fear of severe punishment was not a deterrent. Moreover, the law no longer allowed for condemning an offender to life imprisonment.^86^ As Hájková informed *Vlasta* readers, there were only two options – a fifteen-year imprisonment or the death penalty. Hájková suggested a third option proven abroad – the institutions: “Institutions (*ústavy*) in which the offender is reliably isolated from the rest of the world, but at the same time is professionally treated: psychotherapy, work, hormone treatment, in some cases even castration. These are expensive institutions [...] However, the results have been excellent: those who have been released from such institutions after several years almost never repeat their sex crimes.”^87^ Hájková was annoyed that “the Ministry of the Interior claims that such an institution should be a healthcare institution, the Ministry of Health has no means to build and sustain such an institution, and the Ministry of Justice lacks the competencies to run any facilities of this kind.”^88^

The letter writers also suggested isolation, treatment, and/or castration as possible solutions. The mother of a seven-year-old girl – who had been harassed twice by a stranger – described the perpetrators as “creatures who in many cases cannot even be described as human, but simply as human monsters.”^89^ Because the letter writers evaluated these offenders as ill, “abnormal,” and “sexually perverted,” they proposed detention centers as the ideal solution. A mother of three children from the village of Rosice u Brna wrote, “I think that these perverts should go to an institution where they can be treated and still be helpful to our society by doing work. Considering that they are not normal, the death penalty would be harsh unless they have someone’s life on their conscience. I know that we do not have such asylums at hand. It may be a financial problem to build them. But everyone would welcome a collection for such a purpose.”^90^ People observed the recidivism of these offenders with concern, and repeatedly suggested permanent solutions such as executions or at the very least detention centers and castration, as prison sentences alone did not deter perpetrators from committing further crimes.

## Reactions of Experts to the “Dark Corners” Series

In the third part of the “Dark Corners” series, *Vlasta* published the reactions of four experts. It was another important moment when, after using readers’ letters to support her argument, Hájková devoted space in *Vlasta* to the newly emerging consensus among experts about ineffectiveness of the punishments and the need to isolate the perpetrators from society. These reactions included letters from Olga Štolová (a medical doctor and the Head of the Department of Women and Child Care, Ministry of Health), Jan Škoda (Ministry of Education and Culture), Vladimír Hladil (jurist, Legislative Department of the Ministry of Justice), and Kurt Freund (a psychiatrist and sexologist, Psychiatric Research Institute, Praha-Bohnice). Štolová agreed with Hájková and her approach to sexual delinquents who should be medically treated and permanently isolated from the rest of the society. Štolová, on behalf of pediatricians and child psychiatrists, emphasized the need to protect children and rejected “hesitant attitudes toward the offenders.”^91^

Hladil argued that sexually perverted (*pohlavně úchylní*) individuals defended themselves by insisting that they could not suppress their inclination. Their mental disorder – or insanity, according to Hladil – called for a different solution than imprisonment: protective treatment. But as Hladil emphasized, even if the court imposed such protective treatment, it might not work as it usually lasted only for a short period of time, as exemplified by Alois Polách. Polách had been sentenced to protective treatment from which he was released after eight months when psychiatrists declared that, “the treatment succeeded in cultivating moral barriers and he can be considered cured.”^92^ One year after his release, Polách raped and murdered a four-year-old girl. Hladil pointed out that the disagreement among experts regarding the diminished sanity of a perpetrator of sexual crime prevented institutionalizing such a perpetrator. He reassured the readers that,

the Ministry of Justice is currently taking steps to ensure a consistent and, more importantly, correct assessment of the nature of sexual deviance in scientific forums, on which the determination of further action depends. Once this problem has been resolved, the Ministry, in cooperation with all those concerned, intends to advocate that the protective treatment of sexually deviant persons should be carried out in a new way and in conditions that ensure that they do not re-offend.^93^

The protective treatment – if needed – could last until the end of the offender’s life.

The sexologist Freund emphasized the ineffectiveness of the punishments for sexual deviations (*sexuální úchylky*), suggesting that preventing such crimes was possible. “First and foremost, the idea is that such cases should only be evaluated by psychiatrists or sexologists who are involved predominantly in this area. Only these doctors can determine the measures, the treatment, and its duration. Another condition for progress in this area would be the establishment of a special hospital (*speciální léčebna*) where treatment could be carried out, and patients would be isolated at the same time.”^94^ Among other measures, Freund mentioned hormonal treatment, temporary or permanent isolation, and castration even without the offender’s consent.

## Debates Intensify and Complex Treatment System is Established

Papež’s case re-opened the question of assessing deviants’ ability to control their sexual urges and not to act on them. In May 1967, a round-table discussion organized by the committee for psychiatric sexology was devoted to the topic, attended by a Supreme Court judge, psychologists, and sexologists.^95^ Although the opinions of the participants were not unified, the idea prevailed that, “society imposes a task on those people [deviants] that is difficult to accomplish: long-term to lifelong abstinence from sexual, and for them specific, needs.”^96^ Experts suggested assessing a deviant’s capacity to control his pathological yearnings by determining his ability to compensate for deviant activities and evaluating “a structure of his inhibitory psychic mechanisms.”^97^ Some opined that the likelihood of repressing one’s deviant desires rises with intellect – because only with sufficient mental capacities could a deviant fully understand how dangerous his sexual offenses would be for society. The experts agreed to differentiate sexual delinquents into either deviant or non-deviant categories, a crucial step for establishing their sanity (*příčetnost*) and determining their punishment and treatment. The deviant was seen as pitiable because he had no legal or socially acceptable opportunity to satisfy his specific sexual urges – and as such, his sanity was deemed to be diminished. The non-deviant, on the other hand, was seen as in fact degenerate. He could fully please his sexual appetite in a conventional (i.e., heterosexual and consensual) way, but transgressed sexual and social boundaries anyway. Consequently, if the delinquent were not diagnosed as deviant, he would be judged more severely, unless his sanity and/or recognition skills were not otherwise compromised (e.g., by low intellect or psychological diagnosis).^98^

The conclusions of the round-table discussion were further reinforced by a large body of research on pedophilic offenders that was published by Prague sexologists in the late 1960s. Prior to that, only three articles on pedophilia had ever been published in Czechoslovak medical journals, and knowledge about sex offenders against children was rather scarce. The group of experts from the Sexological Institute in Prague carried out extensive research on pedophilic offenders between 1964 and 1967, exploring a sample of 100 men who had been prosecuted or convicted for sexual contact with children younger than fifteen years old and in whose trials sexologists had served as forensic experts.^99^ Beginning in 1968, the authors published a series of articles based on the study. The experts paid attention to various aspects of the delinquent personality. They examined age, education and intelligence, marital status and marital satisfaction, personal sexual history, the family constellation in childhood, other criminal activity, and somatosexual findings (i.e., measurements of different body parts of the offenders, including penis size). The research results were also presented outside of the pages of medical journals, for example in the criminological press. Thus, criminologists were able to read for the first time about how to recognize and evaluate a pedophilic offender.^100^ More specifically, sexologists informed the expert readers of criminological journals about the characteristics of delinquents’ personalities and about the circumstances of pedophilic crimes, such as where and how the offenders established pedophilic relationships, how children reacted, and how long it took on average for the crime to be discovered.

Based on their research findings, the Prague sexologists summed up the basic diagnostic typology of sex offenders. They stressed that the main task of a forensic expert was to distinguish whether the perpetrator was deviant (“person sexually aberrant in the full sense of the word”) or non-deviant (“i.e., person who is not aberrant permanently but still manifests pedophilic behavior”).^101^ They described a pedophilic deviation as a “sexual psychopathy” – an “unchangeable morbidity” that is “rooted in personality” and as such “hardly (or not at all) influenced by treatment.”^102^ They expressed their conviction that, “During the life of a psychopathic individual, the symptoms always worsen (into his further and ‘deeper’ psychopathy, e.g., due to external stimuli). Improvement is expressed only rarely.”^103^ Sexologists suggested that such a perpetrator would be diagnosed with pedophilic deviation resulting in his diminished soundness of judgment, with the prospect of life-long treatment, eventually combined with detention.^104^ By contrast, in cases of non-deviant pedophilic perpetrators, their pedophilic activity was characterized as “a situationally conditioned sexual stimulation, excitement, and satisfaction.”^105^ This non-deviant group consisted of offenders with personality degradation, who “succumbed to antisocial instincts, perversion (*zvrhlosti*), or indulgent curiosity,” and also of socially unskilled men who were unable to find a suitable partner and who sexually abused a child or children because they were easier to manipulate than the mature companion these men would have otherwise preferred.^106^ The experts briefly clarified that, “Rationally, the forensic evaluation of these non-deviant cases should remove the suggestion of mitigating circumstances, if the diminished soundness of judgment did not come from other psychopathological causes such as imbecility, dementia, etc.”^107^ Again, the question of offenders’ intelligence came to the fore, as the experts related the ability to mask the sexually delinquent behavior to the intelligence of the perpetrator. The authors expected that more intelligent people were better able to cover or substitute for their pathological sexual desires than less clever individuals.^108^ The hypothesis was confirmed by the research. According to the sexologists, none of the “weak-minded people,” who represented a significant proportion of the participants in the research, tried to suppress their aberrant sexual tendencies.^109^

In the late 1960s, Czechoslovak experts agreed on the diagnostic typology of sex offenders — the differentiation between sexual deviants and non-deviant sex offenders — by which the treatment of the delinquents was to be distinguished. Subsequently, treatment regulations for sexual deviants were gradually implemented in the early 1970s. Prior to that, there had been no official treatment provisions for sex offenders. In the early 1970s, the Ministry of Health published several methodical treatment regulations for sex offenders.

First, ministerial decrees in 1971 and 1972 standardized the rules for performing surgical castration.^110^ Therapeutic castration had been legal since 1966, but its practice was not medically specified until 1971. A list of indications was simultaneously issued that included a diagnosis of (further unspecified) “serious sexual deviation” as an indication for castration.^111^

Second, the Ministry of Health published a methodical measure about “The execution of protective treatment ordered by the court” (legislated under the aforementioned §72 of Criminal Act No. 140/1961 Coll.) to ensure its uniform procedure.^112^ The measure ordered psychiatric facilities to inform the court about every change for an individual in court-ordered treatment (*osoba ve výkonu léčení*), specifically when the protective treatment was to be changed from inpatient to outpatient treatment, or when the person was about to be released from inpatient residence. Psychiatric facilities were also obliged to immediately inform the district court if an offender refused to undergo protective treatment, did not cooperate with the therapy ordered, or absconded from the facility. This was to enable better cooperation between courts and experts, which had been lacking in the past.

Third, the Ministry of Health issued a measure for “The dispensary care of sexual deviants” in 1974 that further specified the regulations for their court-ordered treatment (COT).^113^ The measure officially declared that “the execution of the sentence alone cannot change sexual deviants.”^114^ Therefore, outpatient or inpatient COT was to be ordered for them in addition to imprisonment. It also stipulated that in cases of “more serious sexual deviations,” the protective COT should be inpatient and long-term.^115^ Notably, the regulation referred not only to court-ordered protective treatment after committing a sexually motivated offense, but also it called for the active identification of sexual deviants in the population, regardless of whether they had committed a sexually motivated crime. It explicitly emphasized that, “When providing therapeutic and preventive care for sexual deviants, it is important to actively search for the affected persons [sexual deviants], their comprehensive examination, planned treatment, and continuous monitoring of their health status.”^116^ The measure pointed out that §23(4) of Act No. 20/1966 Coll. allowed medical professionals to “perform examinations and treatment procedures without the patient’s consent and, if the nature of the illness [sexual deviation] so required, to admit the patient to inpatient care.”^117^ Therefore, if a sexual deviation was diagnosed in a man but he refused to undergo appropriate sexological treatment, he could be forced to undergo it even without his consent. In this way, the importance of early detection of persons exhibiting “sexually deviant behavior” was requested by the state, which was to guarantee the effective prevention of behavior that could potentially threaten society.

In the very same year when the “Dispensary care for sexual deviants” measure came into force, the first psychiatric ward started to provide specialized inpatient sexological treatment for sexual delinquents.^118^ The sexological ward in Horní Beřkovice was the first of its kind in Czechoslovakia. The doctors created a complex regime treatment for sex offenders, which became a model for the therapy of sexual delinquents in Czechoslovakia and was replicated in other newly opened sexological protective treatment departments around the country in the following decades. The goal of the treatment was to re-socialize sex offenders and turn them into sexually normal persons eligible to be released from the inpatient facility without posing a risk to society.^119^ A specialized and comprehensive system of protective treatment for sexual delinquents was finally established, designed to treat sexual delinquents and prevent recidivism of sexologically untreated sex offenders^120^ – as had been the case with Papež.

## Conclusion

In this article, we show how new kinds of categories – deviant and non-deviant sex offenders against children – emerged as a result of shifting networks of expertise and how treatments, together with isolation from society, were devised as a part of the “network of expertise,” which included agents, concepts, and institutional and spatial arrangements. We argue that in addition to experts (sexologists, psychiatrists, and eventually criminologists), another important agent played a role: the general public, as mediated by the editor of a widely read magazine. We analyze how the concepts imposed on sex offenders changed regarding their ability for self-control and their soundness of judgment, their normalcy or deviance, their prospects for change, and their danger to society. Stemming from this conceptual change, we demonstrate how new institutional and spatial arrangements were put in place. As a result of state-issued guidelines, an institution of protective treatment, which had been conceived by experts and called for by the public, materialized in specialized sexological wards of psychiatric hospitals. Pointing out the shifting conceptual apparatus together with institutional arrangements, we conclude that new frameworks emerged of sexual deviants, delinquents, and patients with court-ordered treatment, and that the understanding of who these people were and what should be done with them changed as a result.

How does gender enter into the story about how child sex offenders became seen as dangerous criminals in Czechoslovakia? At first glance, there is a relative dearth of women: the perpetrator in the breakthrough case was male, and so was his victim. Most of the experts making binding pronouncements over who was a sex offender and how to deal with him were also men. Yet we see gender as important on at least two levels: among the agents driving change and among the people whose interests were invoked in the shift.

On the first level, we see the role of the female editor of the women’s magazine *Vlasta*, Věra Hájková, as crucial. Hájková recognized the need to publicly address the sexual murder of a child and did so using the window of greater journalistic freedom in the mid-1960s in Czechoslovakia. She invited readers to react to this crime, selected the letters to be published, and invited experts to respond. As a result of her agency, people’s voices calling for a specific measure of protective treatment received a lot of space, and her invited experts were those whose ideas about the proper treatment for sex offenders had just coalesced around sexological protective treatment.

On the second level, the interest invoked was that of vulnerable children. Since the readers of *Vlasta* who penned the letters were both men and women, both parents and people who did not disclose their parental status, we cannot conclude that the reactions in *Vlasta* qualify as maternal activism. Yet the interests of children, which fueled the public outcry and legitimized the proposed measures, are symbolically connected with women as mothers and coded as feminine. The network of expertise was thus importantly driven by gendered concerns.

We point out a development that may seem paradoxical from the point of view of liberal democracies. In those Western countries, the 1960s are thought of as an era of liberalizing all kinds of stifling norms (sexual, gender, familial, and others) and a time of deinstitutionalizing psychiatric care. The realities of 1960s Czechoslovakia were contrary in many respects. It was a time when family gained a more normative traditional meaning and sexual transgressions became more policed. In the 1950s, expert and state-imposed discipline of these kinds of people was rather absent; by the 1970s, they were being diagnosed, forensically labelled, and placed in special wards of psychiatric institutions. The 1960s in Czechoslovakia were thus a time when the outlook shifted to institutionalization. Moreover, this disciplining shift was made possible to a large extent by the relative freedom gained by the media, however briefly, and facilitated by the voices of ordinary people who do not typically have much traction in authoritarian societies. The dark corners of human conduct were thus disciplined by a strengthened network of expertise that built on people calling for more safety for children. This network outlived state socialism by many decades, and the system of complex sexological protective treatment, with only a few changes, is still in operation in the Czech Republic today.

## Footnotes

1 Letter 14 July 1966, cited in the documentary “Hrdelní zločiny: Bez slitování” [Capital Crimes: No Mercy] produced by Czech Television in 2001, available at https://www.youtube.com/watch?v=seDIqcxBtP4.

2 Gil Eyal, “For a Sociology of Expertise: The Social Origins of the Autism Epidemic,” *American Journal of Sociology* 118 (2013): 863.

3 Ibid.

4 Benjamin Ziemann, Richard Wetzell, Dirk Schumann, and Kerstin Brückweh, “Introduction: The Scientization of the Social in Comparative Perspective,” in *Engineering Society: The Role of the Human and Social Sciences in Modern Societies, 1880-1980*, ed. Kerstin Brückweh, Dirk Schumann, Richard Wetzell, and Benjamin Ziemann (Basingstoke: Palgrave MacMillan, 2012).

5 Ian Hacking, “Making Up People,” in *Reconstructing Individualism: Autonomy, Individuality, and the Self in Western Thought*, ed. Thomas C. Heller, Morton Sosna, and David E. Wellbery (Stanford: Stanford University Press, 1986), 228.

6 Viola Singer, J. Smolíková, E. Šádková, and Roman Útrata, “Režimové oddělení pro léčbu sexuálních delikventů,” *Československá psychiatrie* 73 (1977): 41–42.

7 Kate Davison, “Cold War Pavlov: Homosexual Aversion Therapy in the 1960s,” *History of the Human Sciences* 34 (2021): 89–119.

8 Sarah Marks, “Ecology, Humanism and Mental Health in Communist Czechoslovakia,” in *Psychiatry in Communist Europe*, ed. Mat Savelli and Sarah Marks (Basingstoke: Palgrave MacMillan, 2015), 135.

9 Ibid., 136.

10 Davison, “Cold War Pavlov,” 96.

11 Ibid., 108.

12 Ibid. See also Jan Seidl, ed., *Od žaláře k oltáři: Emancipace homosexuality v českých zemích od roku 1867 do současnosti* (Brno: Host, 2012).

13 Kateřina Lišková, “‘Now You See Them, Now You Don’t’: Sexual Deviants and Sexological Expertise in Communist Czechoslovakia,” *History of the Human Sciences* 29 (2016): 49–74; Kateřina Lišková, *Sexual Liberation, Socialist Style: Communist Czechoslovakia and the Science of Desire, 1945–1989* (Cambridge: Cambridge University Press, 2018), 228–254.

14 Lišková, “‘Now You See Them, Now You Don’t,’” 49.

15 In 1974, the Ministry of Health issued the “Guidelines of Sexology, Methodological Measures,” which officially defined sexology as a separate branch of medicine. “Koncepce sexuologie, metodická opatření č. 37/1974,” *Věstník MZ* no. 22–24 (1974): 232–233.

16 Kateřina Lišková and Andrea Bělehradová, “‘We Won’t Ban Castrating Pervs Despite What Europe Might Think!’: Czech Medical Sexology and the Practice of Therapeutic Castration,” *Medical History* 63 (2019): 330–351.

17 Kateřina Lišková and Lucia Moravanská, “From Class Origins to Individual Psychopathology: Spousal Murder According to State Socialist Czechoslovak Criminology,” *History of the Human Sciences* 35 (2022): 237–259.

18 Ibid., 238.

19 Ibid., 251.

20 Střelec references both the new Socialist Constitution of 1960 and the Criminal Code of 1961. Jakub Střelec, “Psychopaths as the ‘New Danger’ to Socialist Society: Forensic Psychiatry, Criminology, and Crime in the Czechoslovakia of the 1960s,” from Shaping the Socialist Self? The Role of Psy-Sciences in Socialist States of the Eastern Bloc Workshop, Prague, Czech Republic, November 2020, 11.

21 Ibid., 9.

22 Greg Eghigian, *The Corrigible and the Incorrigible: Science, Medicine, and the Convict in Twentieth-Century Germany* (Ann Arbor: University of Michigan Press, 2015).

23 Ibid., 96.

24 Agnieszka Kościańska, *Gender, Pleasure, and Violence: The Construction of Expert Knowledge of Sexuality in Poland* (Bloomington: Indiana University Press, 2020), 159–183; Agnieszka Kościańska, “Gender on Trial: Changes in Legal and Discursive Practices Concerning Sexual Violence in Poland from the 1970s to the Present,” *Ethnologia Europaea* 50 (2020): 111–127.

25 The Czechoslovak criminal law recognized diminished sanity (*zmenšená příčetnost*) as a distinct legal category beyond the binary of sanity and insanity. The Criminal Code of 1950 (Zákon č. 86/1950 Sb.), §21a used the term *zmenšená příčetnost* as a mitigating circumstance. In the Criminal Code of 1961 (Zákon č. 140/1961 Sb.), §25 and §32 discussed the term and its consequences for sentencing. The Criminal Code of 1961 particularly addressed the cases of offenders with diminished sanity (most commonly those with mental disabilities or severe psychopathy) who posed significant social and rehabilitative challenges. When dealing with offenders who committed crimes under diminished sanity, the courts could both adjust the sentence and mandate protective treatment, as mere reduction of punishment without treatment could weaken both the rehabilitation of the offender and the protection of society.

26 Ferdinand Knobloch and Jiřina Knoblochová, *Soudní psychiatrie pro právníky a lékaře* (Praha: Orbis, 1957), 55.

27 Ibid., 130.

28 For homosexuality, they specifically claimed that it was incurable, which they might have known from the work of their Psychiatry Clinic colleague Kurt Freund. For more on Freund’s trailblazing study of male homosexuality, see Seidl, *Od žaláře k oltáři*; Lišková, “‘Now You See Them, Now You Don’t’”; Davison, “Cold War Pavlov.”

29 The 1965 re-edition of this book devoted more space to sexual deviance, including case studies.

30 Ivan Horvai, “Psychopatie,” in *Speciální psychiatrie*, ed. Zdeněk Mysliveček (Praha: Státní zdravotnické nakladatelství, 1956), 116.

31 Ibid.

32 Ivan Horvai, “K některým praktickým otázkám problematiky příčetnosti,” in *Problémy psychiatrie v praxi a ve výzkumu*, ed. Stanislav Krákora (Praha: Státní zdravotnické nakladatelství, 1957), 71. According to the Criminal Code of 1950, sexual abuse was defined as sexual intercourse or other sexual contact with a person younger than fifteen years old, and could be punished by one to ten years of imprisonment or ten to twenty years of imprisonment if the result was a) serious injury to the child, or b) of a particularly aggravating circumstance. Eventually, there could be verdict of a life imprisonment as well, if the offense resulted in the death of a victim. §239 zákona č. 86/1950 Sb.

33 Knobloch and Knoblochová, *Soudní psychiatrie pro právníky a lékaře*, 41.

34 Ibid., 92.

35 Ibid., 19.

36 Ibid., 94.

37 Ibid., 212.

38 Ibid.

39 Horvai, “K některým praktickým otázkám problematiky příčetnosti,” 72.

40 Ibid., 71.

41 § 124 odst. 1, Zákon č. 87/1950 Sb. o trestním řízení soudním (trestní řád).

42 Ibid., 70.

43 Lišková, “‘Now You See Them, Now You Don’t.’” During the 1950s and early 1960s, sexologists treated 127,676 patients in the Sexological Institute. At the center of sexological interest were infertility problems (45% of patients) and sexual dysfunctions (35% of the cases); only 4% of the patients of the Institute were diagnosed with “sexual aberrations.” Vladimír Barták, “Československá sexuologie a dvacet let rozvoje Sexuologického ústavu,” *Časopis lékařů českých* 104 (1965): 530. Cases of homosexuality were also listed among the “aberrations” at the time, because homosexuality was still criminalized (this changed with the Criminal Code of 1961). Considering the context, we can assume that there would be significantly fewer cases of sexual deviants captured by the statistic if homosexuals were excluded. Indeed, this logic was confirmed by chief sexologist Josef Hynie, who registered only 2% of deviance in 1966, i.e., after the legalization of homosexuality. Josef Hynie, “Problémy sexuologické péče v ČSSR,” *Československá gynekologie* 31 (1966): 561.

44 Karel Nedoma, “Některé poznatky z posuzování sexuálních delikventů z hlediska sexuologa,” *Československá psychiatrie* 54 (1958): 164–169.

45 Karel Nedoma and Kurt Freund, “Otázka příčetnosti a nápravných opatření u sexuálních delikventů,” *Československá psychiatrie* 55 (1959): 264–269.

46 Ibid., 264.

47 Ibid.

48 Ibid., 265.

49 Ibid.

50 Knobloch and Knoblochová, *Soudní psychiatrie pro právníky a lékaře*, 212. For details see Lišková and Bělehradová, “‘We Won’t Ban Castrating Pervs Despite What Europe Might Think!’”

51 Fond NAD 314 Ministerstvo zdravotnictví, Praha. Kt. Psychiatrie, Dietetika, Chirurgie. Soubor, “Psychiatrie 1960–1964.” (Zpráva o výsledcích prověrek psychiatrických léčeben).

52 Ibid. The report is not concerned with specific medications used for specific diagnoses but rather with the inefficient ways medication was ordered, the faulty ways it was stored, missing or incomplete logs about its usage and effects, medication being prescribed by personnel other than medical doctors, etc.

53 Ibid. (Zpráva o situaci a úkolech na úseku psychiatrické péče v ČSSR).

54 Ibid. (Zpráva o výsledcích prověrek psychiatrických léčeben).

55 Ibid. (Zpráva o situaci a úkolech na úseku psychiatrické péče v ČSSR).

56 Ibid. (Zpráva o stavu psychiatrické péče v ČSSR).

57 Nedoma and Freund, “Otázka příčetnosti a nápravných opatření u sexuálních delikventů.”

58 Fond NAD 314 Ministerstvo zdravotnictví, Praha. Kt. Psychiatrie, Dietetika, Chirurgie. Soubor, “Psychiatrie 1960–1964.” This meeting was also reported on in the medical press. See Jiří Roubíček, “Zpráva o odborné činnosti, sjezdech a pracovních schůzích v roce 1960,” *Československá psychiatrie* 57 (1961): 133–139.

59 Ibid. Historically, castration served as a means of punishment. As Herzog showed, castration was used during WWII to punish homosexual behavior, alongside “longer prison and workhouse sentences” and “concentration camp sentences.” Dagmar Herzog, *Sex After Fascism: Memory and Morality in Twentieth-Century Germany* (Princeton: Princeton University Press, 2005), 89–90. Castration and imprisonment were also common penalties for homosexual behavior of men in the Netherlands in the decades following WWII. Dagmar Herzog, *Sexuality in Europe: A Twentieth-Century History* (Cambridge: Cambridge University Press, 2011), 119; Roland M. Bertens, “Zedendelinquentie en chirurgische castratie,” *Nederlands Tijdschrift voor Geneeskunde* 162: 22 (2018). Herzog pointed out that the first castration law in the world was introduced in Denmark in 1929 and was often applied to sex offenders and mentally disabled people. Herzog, *Sexuality in Europe*, 82. Indeed, Czechoslovak experts were inspired by Danish and Swiss experts when they pondered and later proposed the legalization of surgical castration as a treatment for dangerous sexual deviants during the 1960s. Nedoma and Freund, “Otázka příčetnosti a nápravných opatření u sexuálních delikventů.” This suggests that castration was considered a compelling measure in cases of dangerous sexual deviants or abnormality where no other treatment seemed sufficiently effective. However, a more detailed analysis of castration policies in an international comparison (also considering different political systems in the countries) is beyond the scope of our article.

60 Fond NAD 314 Ministerstvo zdravotnictví, Praha. Kt. Psychiatrie, Dietetika, Chirurgie. Soubor, “Psychiatrie 1960–1964.”

61 Ibid.

62 Ibid. (Výkon ochranného léčení v psychiatrických léčebnách – nedostatky v hlášení).

63 §72 zákona č. 140/1961 Sb., trestní zákon.

64 §25 zákona č. 140/1961 Sb., trestní zákon.

65 Ferdinand Knobloch and Jiřina Knoblochová, *Soudní psychiatrie pro právníky a lékaře* (Praha: Orbis, 1965), 10.

66 Ibid., 34.

67 Karel Nedoma, “Adaptační léčba sexuálních deviací u mužů,” *Československá psychiatrie* 60 (1964): 238.

68 Ibid.

69 Ibid.

70 Ibid.

71 §27 zákona č. 20/1966 Sb., o péči a zdraví lidu.

72 Jan Raboch and Karel Nedoma, “Indikace testikulární pulpektomie u sexuálních delikventů,” *Československá psychiatrie* 66 (1970): 152–157.

73 The description of the crime and Papež’s past are based on the Czech TV documentary Hrdelní zločiny (2001) and http://kriminalistika.eu/muzeumzla/papez/papez.html.

74
*Vlasta* was published between 1945 and 1989 by the women’s organization. In 1966, *Vlasta* was published in 630,000 copies; by 1967, there were 660,000 copies weekly and around three million readers. Even with this high number of copies, it was unable to meet the demand. For comparison, the average publication for magazines in Czechoslovakia was 200,000 copies. Even though *Vlasta* was a women’s magazine, it was also read by men, often by whole families. Fond 360 Československý svaz žen – ústřední výbor, Praha. Kt. 65. “Podklady pro rozbor časopisu Vlasta.” *Vlasta* was, after *Rudé pravo*, the most-read periodical in Czechoslovakia. Jacqui True, *Gender, Globalization, and Postsocialism: The Czech Republic after Communism* (New York: Columbia University Press, 2003), 43.

75 Jan Stach and Miroslav Kučera, *Popravou život končí (sedmnáct zastavení se smrtí)* (Praha: Police history, 2009).

76 Hájková was the editor of *Vlasta* who focused on children. She wrote about infant institutions, weekly nurseries, kindergartens, children’s homes, etc., and she advocated for the establishment of SOS children’s villages in Czechoslovakia. Hájková also noted that the sexual education of children was insufficient and practically impossible for small children.

77 Věra Hájková, “Temné kouty,” *Vlasta* 20: 40 (1966): 4.

78 We cannot speak about maternal activism, as the letters that were sent regarding the “Dark Corners” series and the cases of sexual crimes against children came from the wider general public.

79 Jan Rychlík, *Československo v období socialismu: 1945-1989* (Praha: Vyšehrad, 2020), 167.

80 Hájková, “Temné kouty.”

81 Věra Hájková, “Ohlas na Temné kouty,” *Vlasta* 20: 49 (1966): 11.

82 Ibid.

83 Fond 360 Československý svaz žen – ústřední výbor, Praha. Kt. 19. “BULLETIN /Ohlasy na TEMNÉ KOUTY/,” 3.

84 This writer is probably reacting to the sexual murder of a four-year-old girl committed by Polách. Ibid., 3–4.

85 Ibid., 5.

86 In 1961, the law was changed, and the penal rates specified in the paragraph devoted to “sexual abuse” (*pohlavní zneužívání*) were reduced. The crime could be newly punished by 1) one to eight years of imprisonment for having sexual intercourse (or another form of sexual contact) with a person younger than 15 years; 2) two to ten years of imprisonment if the offender committed the act against a person under their supervision, taking advantage of their dependence; 3) five to twelve years of imprisonment if the result was serious injury to a child’s health; or 4) ten to fifteen years if the offense resulted in the death of a child. §242 zákona č. 140/1961 Sb.

87 Věra Hájková, “Temné kouty,” *Vlasta* 20: 40 (1966): 4–5.

88 Ibid., 5.

89 Fond 360 Československý svaz žen – ústřední výbor, Praha. Kt. 19. “BULLETIN /Ohlasy na TEMNÉ KOUTY/,” 6–7.

90 Ibid., 9–10.

91 “Temné kouty. Tentokrát názory odborníků,” *Vlasta* 20: 50 (1966): 7. More about the attempts to introduce systematic sexual education in late-socialist Czechoslovakia in Kateřina Lišková, Natalia Jarska, and Gábor Szegedi, “Sexuality and Gender in School-Based Sex Education in Czechoslovakia, Hungary and Poland in the 1970s and 1980s,” *History of the Family* 25 (2020): 550–575.

92 “Temné kouty. Tentokrát názory odborníků,” 7. Hladil did not specify Polách’s previous sentence. According to an article in the daily *Rudé právo*, Polách had been prosecuted twice for the sexual abuse of two girls and one boy, and the attempted rape of a 70-year-old woman. “Trest smrti vrahovi,” *Rudé právo* 7: 15 (1966): 4.

93 “Temné kouty. Tentokrát názory odborníků,” 7.

94 Ibid.

95 Jiří Mellan, “Ovládací schopnost sexuálních deviantů,” *Československá psychiatrie* 64 (1968): 209–211.

96 Ibid., 211.

97 Ibid.

98 Ibid.

99 Karel Nedoma, Jiří Mellan, and Jaroslava Pondělíčková, “Sexuální chování a jeho vývoj u pedofilních mužů,” *Československá psychiatrie* 65 (1969): 92–98; Jaroslava Pondělíčková, Jiří Mellan, and Karel Nedoma, “K osobnosti pedofilních delikventů,” *Československá psychiatrie* 65 (1969): 155–158.

100 Karel Nedoma, Jiří Mellan, Jaroslava Pondělíčková, and J. Löschner, “Sexuální delikty na dětech,” *Kriminalistický sborník* 12 (1968): 170–174; Jaroslava Pondělíčková, Jiří Mellan, and Karel Nedoma, “K problému pedofilní delikvence,” *Československá kriminalistika* 1 (1968): 94–99.

101 Pondělíčková, Mellan, and Nedoma, “K problému pedofilní delikvence,” 97.

102 Ibid.

103 Ibid., 98.

104 Although the authors spoke about detention, no institution as such existed at that time. Security detention was not founded in the Czech Republic until 2009. Pavel Vantuch, “Zabezpečovací detence, její ukládání, výkon, trvání a obhajoba obžalovaného,” *Trestní právo* 15: 9 (2010): 5–13.

105 Pondělíčková, Mellan, and Nedoma, “K problému pedofilní delikvence,” 99.

106 Ibid.

107 Ibid.

108 Ibid., 95.

109 Ibid.

110 “Léčebné zákroky u intersexuálů, transsexuálů a sexuálních deviantů, zprávy a sdělení,” *Věstník MZ* 1–2 (1971): 4–5; “Provádění sterilizace, závazná opatření 1/1972,” *Věstník MZ* 20: 1–2 (1972): 1–4.

111 “Provádění sterilizace, závazná opatření 1/1972,” 4.

112 “Výkon ochranného léčení uloženého soudem, metodické opatření č. 29/1972,” *Věstník MZ* 10 (1972): 113–114.

113 “Dispenzární péče o sexuální devianty, metodické opatření č. 39/1974,” *Věstník MZ* 22–24 (1974): 236–237.

114 Ibid., 237.

115 Ibid. Which types of sexual deviations were deemed to be “more serious” or how long “long-term treatment” should last was not further specified in the measure.

116 “Dispenzární péče o sexuální devianty, metodické opatření č. 39/1974,” 236.

117 Ibid.

118 Singer et al., “Režimové oddělení pro léčbu sexuálních delikventů.”

119 For more information about the sexological protective treatment regime see Lišková, *Sexual Liberation, Socialist Style*; Lišková and Bělehradová, “‘We Won’t Ban Castrating Pervs Despite What Europe Might Think!’”

120 The fact that (sexological) protective treatment started to be ordered for sex offenders by the court is demonstrated in the *Statistical Yearbook of Crime* (*Statistická ročenka kriminality*). As an illustration, we use the year 1982 (published in 1983), which is the last year in which the data about court-ordered protective treatment were aggregated for the Czech and Slovak parts of the Republic. The number of convicted paedophilic sex offenders (recorded under §242 and 243 as “sexual abuse” in the Yearbook) and the number of treatments ordered for them remained quite stable until the end of 1980s. The presented year thus can be seen as a representative case. In 1982, 1,417 known offenders were prosecuted for the crime of sexual abuse, 13.6% of whom committed the offence while under the influence of alcohol. There were 1,106 persons charged, of whom 471 (i.e. 42.6%) had been previously convicted (recidivists), of whom 151 had been convicted for an act of the same kind and thirteen were labelled as “particularly dangerous recidivists.” In the end, 918 men were convicted, 98 of whom had committed crimes while under the influence of alcohol and four of whom were labelled as “particularly dangerous recidivists.” Of the 918 convictions, 393 offenders were sentenced to non-suspended imprisonment and 495 to suspended imprisonment. Punishment was waived for thirty offenders, but three were given in-patient protective treatment, and one was given protective education (which can perhaps be considered outpatient protective treatment). Thirty-five offenders were sentenced in conjunction with another punishment (which we assume was some kind of inpatient protective treatment). Protective supervision – which we believe to mean outpatient protective treatment – was imposed on twenty-two offenders. Based on the figures given, we cannot say with precision whether the duration of sexual deviant desire and/or the gravity of the offence was indeed the decisive circumstance for the court to order sexological protective treatment; at the same time, we cannot say whether the treatment was sexological or anti-alcohol or anti-toxicogenic. Although it is impossible to triangulate what type of people were placed in a particular type of protective treatment, we can conclude that protective treatment was indeed imposed on sex offenders, and we can perhaps assume that it was mainly recidivists who were placed in some type of protective treatment. Generální prokuratura ČSSR, *Statistická ročenka kriminality 1983* (Praha: SNV, 1983), 93, 277.

